# Influence of Ingestion of Bicarbonate-Rich Water Combined with an Alkalizing or Acidizing Diet on Acid-Base Balance and Anaerobic Performance

**DOI:** 10.5114/jhk/182986

**Published:** 2024-05-17

**Authors:** François Chiron, Claire Thomas, Joffrey Bardin, Florence Mullie, Samuel Bennett, Jérémy Chéradame, Laurine Caliz, Christine Hanon, Eve Tiollier

**Affiliations:** 1 LBEPS, Univ Evry, IRBA, Université de Paris-Saclay, Evry, France.; 2 French Federation of Athletics (FFA), Paris, France.; 3 Laboratory of Sport, Expertise and Performance (SEP), French National Institute of Sport (INSEP), Paris, France.; 4 French Federation of Rugby (FFR), Marcoussis, France.; 5 Research Institute for Sport and Exercise Science (RISES), Liverpool John Moores University, Liverpool, United Kingdom.

**Keywords:** high intensity exercise, anaerobic capacity, metabolic acidosis, HCO_3_^-^ supplementation

## Abstract

During high-intensity (HI) exercise, metabolic acidosis significantly impairs exercise performance. Increasing the body's buffering capacity through training and exogenous intake of alkalizing supplements may improve high-intensity performance. Manipulating water and diet intake may influence the acid-base balance. The aim of this study was to determine the effects of mineral water rich in bicarbonate ions (STY) or placebo water (PLA) on circulating biomarkers and anaerobic performance and to verify whether alkalizing (ALK) or acidizing (ACI) diet would modulate these effects. Twenty-four athletes, assigned either to ALK (n = 12) or ACI (n = 12) diet for four weeks, completed a 1-min rowing Wingate Test in a double-blind and randomized trial after one week of daily hydration (1.5 to 2L/d) with either STY or PLA. Blood samples were taken before and after each test, and urine samples were collected each week. Chronic consumption of bicarbonate-rich water significantly impacted resting urinary pH irrespective of alkalizing or acidizing dietary intake. STY induced a significant increase in blood pH, lactate, and HCO_3_^-^ ion concentration post-exercise compared to PLA. Similar changes were observed when STY was associated with the ALK diet. In contrast, STY combined with the ACI diet only significantly affected urine pH and peak blood lactate compared to PLA (p < 0.05). No effect of bicarbonate-rich water was reported on anaerobic performance (p > 0.05). Our results suggest that consumption of bicarbonate-rich water alters acid-base balance during a warm-up and after HI exercise, could potentiate beneficial effects of an alkalizing diet on the acid-base balance after HI exercise, and reduces the acid load induced by an acidifying diet.

## Introduction

During explosive, high-intensity exercise, performance success is determined by an athlete's ability to maintain a maximal effort ranging from few seconds to a few minutes in duration (Hanon et al., 2019). During high-intensity (HI) exercise, glycolysis contributes significantly to energy production, inducing disturbances in acid-base balance, particularly a decrease in muscle and blood pH and hyperlactatemia. Since metabolic acidosis limits the maintenance of physical activity and performance (Millet et al., 2004), increasing the buffer capacity could enhance HI anaerobic performance and reduce blood metabolic disturbances. This can be achieved either through training (Edge et al., 2006) or by the exogenous supply of buffering agents both acutely (Lancha Junior et al., 2015) and chronically (Chycki et al., 2018). One of the most popular buffering agents is sodium bicarbonate, which has been investigated in numerous studies (Grgic et al., 2021). The bicarbonate is typically provided via supplement with potential side effects, especially gastrointestinal distress. Nonetheless, it is important to note that some athletes may be unable to tolerate direct bicarbonate supplementation, as it can lead to gastrointestinal issues (McNaughton et al., 2016; Ragone et al., 2020). Certain coaches and athletes may not be inclined to embrace the concept of enhancing their buffering capacity through methods like bicarbonate supplementation. The enhancement of buffering capacity through a strategy perceived as more natural might receive approval within the sporting community. There is limited research examining supplementation through the regular consumption of naturally occurring water rich in bicarbonate ions (HCO_3_^-^). Chronic intake (1 week) of mineral water with a pH of around 6 and a concentration of HCO_3_^-^ ions around 1000 mg/L has been shown to have either no effect (Chycki et al., 2017) or increase urinary pH (Brancaccio et al., 2012; Halz et al., 2022), after HI exercise. The lowest intake (1.5 L/day of water containing 981 mg/L of HCO_3_^-^) resulted in increased urine pH (Brancaccio et al., 2012), whereas the highest intake (4–4.5 L/day of water containing 1300 mg/L of HCO_3_^-^) did not (Chycki et al., 2017). An explanation could be the timing between the water ingestion and the urine collection. The urine pH was elevated when the water was ingested during the hours before urine collection (Brancaccio et al., 2012), suggesting that the observed effect might be due to acute administration rather than a chronic adaptation. Although those studies include exercise, performance was not measured, and blood markers of acid-base balance were poorly reported.

Consumption of water containing a high bicarbonate content (4368 mg/L) has favored a rapid restoration of acid-base balance after aerobic exercise and permitted greater residual strength following an isokinetic test (Rieux and Duvallet, 2000). In this study, bicarbonate-rich water was not ingested chronically as previously reported (Brancaccio et al., 2012; Chycki et al., 2017), but only on the day of testing (2 h before exercise until immediately before the last test). Altogether, the few data available highlighted the need for further studies regarding the effect of chronic bicarbonate-rich water intake on acid-base balance and anaerobic performance. It has been suggested that the ingestion of bicarbonate-rich water alongside an alkalizing diet may provide a novel nutritional strategy to influence acid-base balance (Limmer et al., 2018; Remer and Manz, 1995) and improve anaerobic performance (Limmer et al., 2018; Steffl et al., 2021). Combining bicarbonate-rich water with an alkalizing diet could enhance the benefits of increasing buffering capacity and potentially lead to improved performance. In contrast, when combined with an acidifying diet, bicarbonate-rich water may mitigate the negative effects of such a dietary pattern. This approach may offer a solution for athletes who prefer acid-forming foods like meat while minimizing the potential drawbacks of such a diet. As such, evaluating how the diet may modulate the effect of bicarbonate-rich water chronic ingestion on exercise performance warrants investigation.

The present study, therefore, aimed to investigate whether the consumption of bicarbonate-rich water combined with an alkalizing diet could provide an additional buffering effect, potentially delaying the effects of metabolic acidosis and improving performance. We also hypothesized that the consumption of bicarbonate-rich water could mitigate the acid load induced by the acidifying diet, particularly in terms of urinary and blood pH.

## Methods

### Participants

Twenty-four recreationally active men (29 ± 8.8 years, 1.79 ± 0.50 m, 81.8 ± 11.6 kg, VO_2max_ 55.6 ± 9.0 ml/min/kg) volunteered to participate in this study. All participants were informed of the study protocol, their rights, and the associated risks of the intervenion before providing written informed consent. All procedures were approved by the committee CERSTAPS (approval No. 2022-A00644-39; approval date: 01 January 2020) and conducted in line with the Declaration of Helsinki (1964, revised 2001). Participants were excluded from the study if they could not follow dietary and hydration recommendations or upon the occurrence of any injury or unusual fatigue during the 4-week protocol that could cause a decrease in performance during the supramaximal test.

### Experimental Design

**Figure 1:**
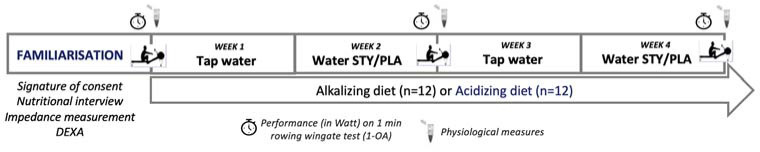
Schematic overview of the study design. During weeks 2 and 4, participants consumed bicarbonate-rich water (STY) or placebo water (PLA) in a cross-over design at a rate of 1.5 to 2 L per day, with habitual hydration resumed during weeks 1 and 3. At the end of each week, a supra-maximal test consisting of a 1-min rowing Wingate test (1-AO) was completed with physiological biomarkers being measured meanwhile. Participants followed also an acidizing (n = 12) or an alkalizing (n = 12) diet for four weeks.

Participants performed two pre-visits (pre- visit 1: consent signature, nutritional interview, urine samples; pre-visit 2: urine and blood samples, rowing maximal test familiarization) (Figure 1). Afterward, participants were assigned to a dietary group at random sampling, either an alkalizing (ALK, n = 12) or an acidizing (ACI, n = 12) diet. A daily check was conducted between our nutritionist and participants to ensure that they were adhering to the nutritional guidelines required by their respective groups. During weeks 2 and 4, participants were instructed to drink 1.5 L to 2 L of either bicarbonate-rich water (STY) or placebo water (PLA) in a cross-over, double-blind, and randomized design as part of their daily hydration. At the end of weeks 2 and 4, a supra-maximal test consisting of a 1 min rowing Wingate Test (1-AO) was completed, with blood biomarkers measured under each condition (STY or PLA).

### Experimental Water

In both weeks 2 and 4, in a double-blind and randomized fashion, participants were instructed to drink 1.5 to 2 L per day of either bicarbonate-rich or placebo water (Table 1) during the first six days of the week. Four bottles of 500 mL were provided daily to each participant. St Yorre water (STY) (Vichy St Yorre, Sources Alma, La Ferrière Bochard, France) is a naturally carbonated water rich in minerals (4774 mg/L containing 4368 mg of HCO_3_^-^ ions). The low-bicarbonate water, as placebo (PLA), was provided to participants in identical bottles to STY, but contained regular carbonated water with considerably lower mineral content (Table 1). Habitual hydration was resumed during weeks 1 and 3.

**Table 1:** Composition of St Yorre water and Placebo water.

	St Yorre (STY)	Placebo (PLA)
Calcium (mg·L**^−1^**)	90	93
Magnesium (mg·L**^−1^**)	11	8.1
Sodium (mg·L**^−1^**)	1708	8.8
Potassium (mg·L**^−1^**)	110	2.6
Silica (mg·L**^−1^**)	16	19
Bicarbonate (mg·L**^−1^**)	4368	306
Chloride (mg·L**^−1^**)	322	18
Sulphate (mg·L**^−1^**)	174	7.5
Nitrates (mg·L**^−1^**)	2.5	2
Fluorine (mg·L**^−1^**)	1	0.35
pH	6.6	5.4
Sodium Bicarbonate Equivalent (mg·L**^−1^**)	6015	-
Sodium Chloride Equivalent (mg·L**^−1^**)	528	-

### Dietary Group

Next to a nutritional interview, participants were assigned at random sampling alkalizing or acidizing diet. To maintain single-blind dietary strategies during the protocol, the diet was presented as a vegetarian- or meat-prone diet. Each participant was given detailed written nutritional guidelines and meal plans for the duration of the study. The ALK diet was based on a vegetarian-prone diet (Caciano et al., 2015; Limmer et al., 2018). Participants were required to consume a minimum of three portions of vegetables and four portions of fruit daily. Dairy products (including yogurt, fresh cheese, curd, and milk) and potatoes were permitted.

Conversely, participants were prohibited from consuming meat, fish, and cheese besides fresh cheese and non-whole meal starchy foods. In contrast, the ACI diet was based on a meat-prone diet (Caciano et al., 2015; Limmer et al., 2018). Participants consumed three portions of animal protein (meat, fish, or eggs), three portions of starch, and three portions of dairy including at least one portion of cheese and were required to avoid consuming any bean, vegetable or fruit.

During the entire protocol, a trained dietician monitored dietary intake via a written food diary and accompanying photographs of meals to ensure that participants followed the recommandations. Moreover, the Potential Renal Acid Load (PRAL) (Remer and Manz, 1995) was calculated to verify the characteristic of either the alkalizing or the acidizing diet. The goal for the alkaline-trending diet group (ALK) was to achieve a dietary PRAL ≤ −1 mEq /d, and for the acid-trending group (ACI), a PRAL ≥ 15 mEq/d (Peter et al., 2010).

### Data Collection

#### Urine Analysis

During the 6^th^ day of weeks 2 and 4, participants completed a 24-h urine collection. Urinary pH was assessed using a pH probe (914, Metrohm, Switzerland).

On day 7 of each week, participants reported to the laboratory having consumed the same dietary intake for the 24 h before each visit.

#### 1-AO Test

In the week before the commencement of the study, participants completed a 1-AO which served as a familiarization for the upcoming trials. Participants performed a 10-min, self-paced warm-up (replicated between trials) followed by a maximal 1-AO on a rowing machine (PM5, Concept2, USA) with instructions to complete an “all-out” effort and not to be concerned about pacing strategies. Performance was assessed as maximum (P_max_) and average (P_mean_) power output in absolute (W) or relative to body weight (W/kg) values. Perception of effort (RPE) and the fatigue index in the last 10 s of the test (IDF 50–60 = [P_mean_ 50–60 − P_max_] / P_max_) were also determined.

#### Blood Acid-Base Status

Blood biomarkers, including blood lactate concentration ([La]), blood HCO_3_^-^ concentration ([HCO_3_^-^]), and blood pH, were measured to determine acid-base regulation weekly (day 7) via earlobe-prick samples (20 μL) after the warm-up, immediately after exercise (+0'), then 3 (+3'), 5 (+5'), and 8 (+8') minutes (passive recovery) following the 1-AO. Samples were analyzed via blood gas and electrolyte analyzer (i-STAT, Abbott, USA).

### Statistical Analyses

Statistical analyses were performed with RStudio software (version 3.5.0, Inc. Boston, MA, USA). To assess the effect of water (STY vs. PLA) on acid-base balance and performance, we applied a linear mixed model on the whole population with water as a fixed effect. The model was adjusted for the dietary group as the dietary group was also included as a fixed effect.

After that, two mixed models were created, one for each dietary group, to evaluate to what extent diet modulated the effect of water intake. Within each model, water intake was maintained as the fixed effect, and the random effect was the subject (STY^ALK^ vs. PLA^ALK^ and STY^ACI^ vs. PLA^ACI^). The variables studied were biomarkers of acid-base balance and performance. The characterization of the diet as either acidizing or alkalizing was assessed against the PRAL of the diet. Comparison of PRAL was assessed with Student’s *t* tests either for paired data (STY^ACI^ vs. PLA^ACI^ and STY^ALK^ vs. PLA^ALK^) or independent groups (ACI vs. ALK). The normality of data was checked via the Shapiro-Wilk test before using this parametric test.

The results are presented as means ± standard deviations. The coefficient of the model represents the mean difference when placed in a condition relative to the reference condition in the unit of measurement of the variable studied. A difference was considered significant at *p* < 0.05. Cohen's *d* was calculated, enabling us to categorize the magnitude of the error of estimation as large (0.8), moderate (0.5–0.8), or small (0.5) and to facilitate a more in-depth discussion of our results (Rosenthal and al., 1994).

## Results

### Water Consumption and Bicarbonate Intake

The mean daily volume of water ingestion was 1919 ± 65 mL and 1842 ± 34 mL, with a corresponding bicarbonate ion content of 8381 ± 539 mg and 564 ± 39 mg for STY and PLA, respectively. The resultant relative sodium bicarbonate intake (given that 1 g of sodium bicarbonate contains 726 mg of bicarbonate) was calculated to be 146 ± 22 mg/kg/day and 10 ± 2 mg/kg/day for STY and PLA, respectively.

### PRAL of Dietary Groups

The mean PRAL of ACI and ALK groups was significantly different (ACI: 49 ± 13 mEq/d vs. ALK: −13 ± 14 mEq/d, *p* < 0.001). The mean PRAL of the ALK group was within the fixed range for alkalizing diet (PRAL < 1 mEq/d) and was not different between STY^ALK^ and PLA^ALK^ conditions (STY^ALK^: −12 ± 9 mEq/d PLA^ALK^: −14 ± 18 mEq/d, *p* = 0.81).

The mean PRAL of the ACI group was within the fixed range for acidizing diet (PRAL > 15 mEq/d) and was not different between STY^ACI^ and PLA^ACI^ conditions (STY^ACI^: 51 ± 9 mEq/d vs. PLA^ACI^: 47 ± 15 mEq/d, *p* = 0.29).

### Performance

There was no statistical difference in performance variables during the 1-minute supra-maximal test between STY and PLA water groups.

Absolute P_max_, absolute P_mean_, relative P_mean_ (w/kg), P_max_, the fatigue index, and the RPE were not significantly different between the alkaline diet combined with placebo water and the alkaline diet combined with bicarbonate-rich water (*d* = −0.14, −0.09, −0.15, −0.05, −0.07, −0.27, respectively); and between the acidic diet associated with placebo water and the acidic diet associated with bicarbonate-rich water (*d* = −0.33, 0.14, −0.36, 0.18, −0.28, 0.14, −0.31, respectively).

### Biomarkers of Acid-Base Balance

#### Urinary markers

Urinary pH was significantly higher following the consumption of bicarbonate-rich water (STY) compared to placebo water (PLA) after 3 (STY: 7.06 ± 0.50 vs. PLA: 6.14 ± 0.46; *p* = 0.01; *d* = 1.92) and 6 (STY: 7.397 ± 0.3 vs. PLA: 7.387 ± 0.02, *p* = 0.01; *d* = 1.51) days of consumption.

**Figure 2:**
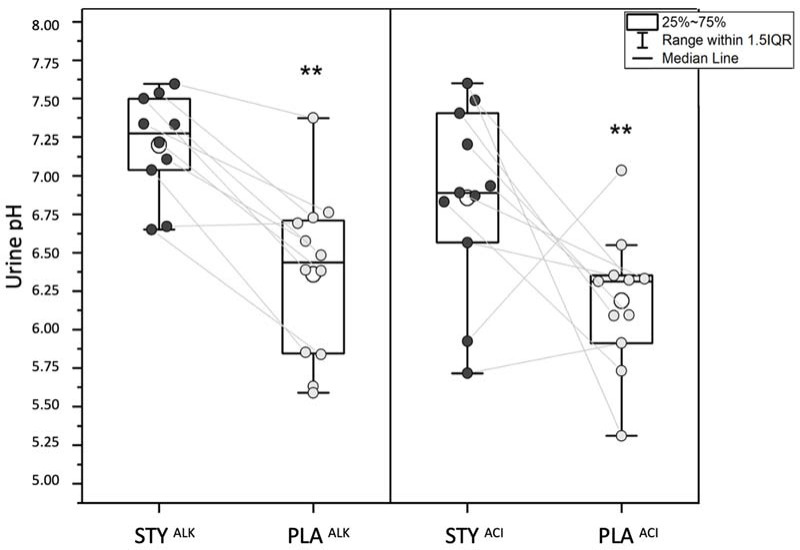
Urinary pH (24-h urine collection on day 6 of the week) after consumption of bicarbonate-rich water (STY) or placebo water (PLA) for 6 days and with an acidizing (ACI) or an alkalizing (ALK) diet. ** Indicates a significant difference with STY (*p* < 0.01).

Urinary pH measured following 24-hour urine collection on the 6^th^ day of water consumption was significantly higher in STY^ALK^ than in PLA^ALK^ (STY^ALK^: 7.20 ± 0.34 vs. PLA^ALK^: 6.36 ± 0.54, *p* = 0.01; *d* = 1.93). It was also significantly higher in STY^ACI^ compared to PLA^ACI^ (STY^ACI^: 6.86 ± 0.60 vs. PLA^ACI^: 6.19 ± 0.45, *p* = 0.01; *d* = 1.28) (Figure 2).

### Effects of Combined Bicarbonate-Rich Water and Diet

#### Blood lactate Concentration

**Figure 3:**
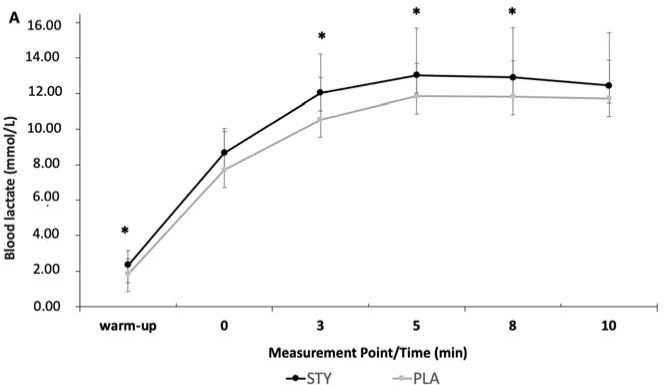
Blood lactate at the end of the 1-min supra-maximal test during passive recovery after consumption of bicarbonate-rich water (STY) or placebo water (PLA) for 1 week. * Indicates a value significantly different from the PLA condition (*p* < 0.05). Warm-up = measurement taken after the 10-min rowing warm-up and before the supra-maximal test; +0 = immediately after the supra-maximal test; +3, +5, +8 and +10: 3, 5, 8 and 10 minutes after the supra-maximal test. The data are presented as means ± standard deviations.

Blood lactate concentrations ([La]) were significantly higher in the STY compared to the PLA group during the warm-up and 3, 5, and 8 min after the supramaximal test. Peak blood lactate ([La]_max_) was significantly higher in the STY (13.4 ± 2.44 mmol/L) compared to the PLA group (12.45 ± 2.65 mmol/L; *p* = 0.035; *d* = 0.37) (Figure 3).

#### Blood Lactate

Peak Blood lactate [La] was significantly higher in the STY^ALK^ compared to the PLA^ALK^ group (*p* < 0.01; *d* = 0.51), as well as after the warm-up and 5 min after the 1-AO during passive recovery (*p* = 0.02; *d* = 0.88 and *p* = 0.04; *d* = 0.65). Regarding ACI, only the peak of [La] was significantly higher in the STY^ACI^ compared to the PLA^ACI^ group (*p* < 0.01).

#### Blood pH

**Figure 4:**
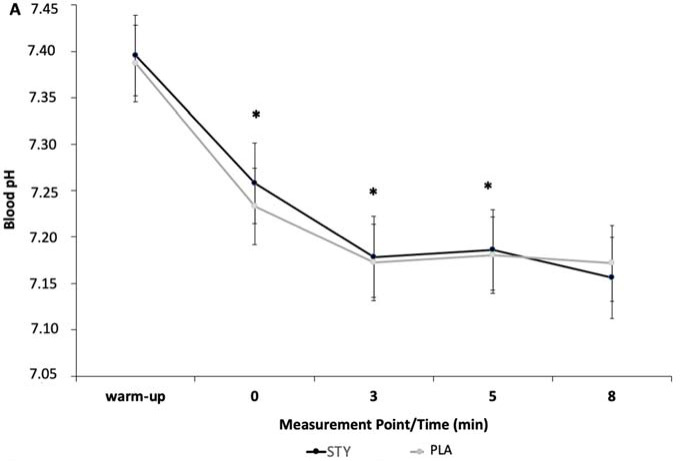
Blood pH after the 1-min supra-maximal test during passive recovery from consumption of bicarbonate-rich water (STY) or placebo water (PLA) for 1. * indicates a significantly different value (*p* < 0.05) of PLA. Warm-up = measurement taken after the 10-min rowing warm-up and before the supra-maximal test; +0' = immediately after the supra-maximal test; +3, +5 and +8: 3, 5 and 8 min after the supra-maximal test. The data are presented as means ± standard deviations.

Blood pH was significantly higher in the STY than the PLA group immediately following, 3, and 5 minutes after the supramaximal test (Figure 4).

Blood pH was significantly different for the STY^ALK^ compared to the PLA^ALK^ group directly after 1-AO (+0') (*p* < 0.01, *d* = 0.79) and 5 min post-exercise (+5') (*p* = 0.02, *d* = 0.22). No significant difference was observed in blood pH in the STY^ACI^ compared to the PLA^ACI^ group.

#### Blood HCO3- Concentration

**Figure 5:**
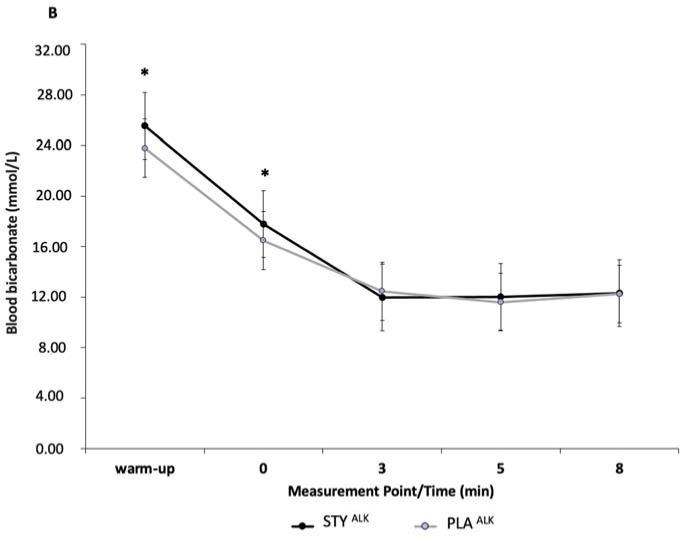
Blood bicarbonate concentration ([HCO_3_^-^]) at the end of the 1-min supra-maximal test during passive recovery from consumption of bicarbonate ion-rich water (STY^ALK)^ or placebo water (PLA^ALK^) for 1 week in combination with an alkalizing diet. * Indicates a significantly different value (*p* < 0.05) of PLA. Warm-up = measurement taken after the 10-minute rowing warm-up and before the supra-maximal test; +0' = measurement taken immediately after the supra-maximal test; +3, +5 and +8: measurements taken 3, 5 and 8 min after the supra-maximal test. The data are presented as means ± standard deviations.

Blood bicarbonate (HCO_3_^-^) concentrations were not significantly different between STY and PLA groups at any timepoint before or after the 1-min supra-maximal test. Peak blood [HCO_3_^-^] was significantly different for the STY^ALK^ compared to the PLA^ALK^ group as well as after the warm-up (*p* = 0.02, *d* = 1.01) and directly after the 1-AO (+0') (*p* < 0.01, *d* = 0.58) (Figure 5). No significant difference was observed for blood [HCO_3_^-^] in the STY^ACI^ compared to the PLA^ACI^ group.

Peak blood [HCO_3_^-^] was significantly different for the STY^ALK^ group compared to the PLA^ALK^ group, as well as after the warm-up (*p* = 0.02, *d* = 1.08) and directly after the 1-AO (+0') (*p* < 0.01, *d* = 0.58) (Figure 5). No significant difference was observed for blood HCO_3_^-^ concentration in the STY^ACI^ compared to the PLA^ACI^ group.

## Discussion

This study was the first to investigate the influence of chronic bicarbonate supplementation via the consumption of water naturally rich in bicarbonate ions on the regulation of acid-base balance and anaerobic performance. Furthermore, to determine whether alkalizing or acidizing diets could modulate the effect of bicarbonate-rich water, we subdivided our population into two groups. We reported that the consumption of naturally carbonated bicarbonate ion-rich water induced a significant increase in urine pH compared to regular carbonated water (*p* < 0.05), irrespective of the diet. Furthermore, despite no water or the diet type effect on anaerobic performance, peak blood lactate concentration during recovery was elevated following bicarbonate-rich water consumption compared to regular carbonated water. Additionally, blood lactate concentration increased comparably in both dietary groups (*p* < 0.05) following bicarbonate-rich water consumption. Post-exercise blood pH was significantly increased with the consumption of bicarbonate-rich water. This effect was maintained following the consumption of an alkalizing diet, whereas it was not with the acidizing diet. Lastly, consuming bicarbonate-rich water alongside an alkalizing diet conferred an additional buffering effect on blood bicarbonate concentration after exercise (*p* < 0.05).

Consistent with the work of Heil et al. (2010), we observed a significant increase in 24-h urine pH after 3 (STY: 7.06 ± 0.50; PLA: 6.14 ± 0.46; *p* = 0.01) and 6 days (STY: 7.02 ± 0.51; PLA: 6.28 ± 0.49; *p* = 0.01) of consumption of mineral water rich in bicarbonate ions in comparison to PLA (*p* < 0.01). Heil et al. (2010) similarly evaluated the impact of alkaline water consumption on acid-base balance and hydration status. However, we reported a faster and more marked effect of bicarbonate ion-rich water consumption on urinary pH, which modified the circulating acid-base balance. Moreover, the alkalinizing effect of bicarbonate-rich water on the urine pH was further altered when combined with alkalizing and acidizing diets. The consumption of bicarbonate-rich water compensated for the acidizing diet-induced acid load on the acid-base balance.

Although no effect of water and the diet type was reported on anaerobic performance, we observed in STY, STY^ALK^, and STY^ACI^ significantly higher values of peak blood lactate concentration after 1-AO compared to PLA, PLA^ALK^, and PLA^ACI^. We also observed in STY, and STY^ALK^ significantly higher values of blood lactate, and pH, after 1-AO compared to PLA and PLA^ALK^. Furthermore, HCO_3_^-^ concentration was augmented when bicarbonate-rich water consumption was combined with an alkalizing diet, characteristic of bicarbonate supplementation (Carr et al., 2011). Indeed, changes in these blood biomarkers, indicative of acid-base balance, have previously been reported following threee weeks of alkaline water intake (Chycki et al., 2018), following the consumption of bicarbonate-rich water during aerobic exercise (Rieux and Duvallet, 2000), and following four days of an alkaline diet (Limmer et al., 2018). Increased peak blood lactate may be explained by higher glycolytic rates, permitted by reduced inhibition of phosphofructokinase activity, an essential enzyme in the control of glycolysis (Robergs et al., 2004). Secondly, blood lactate concentration may be increased due to improved lactate transportation. Specifically, increased activity of transport proteins (membrane co-transporters MCT1 and MCT4) may facilitate the release of muscle lactate into the extracellular environment. The presence of bicarbonate ions, therefore, allows for the removal of extracellular H^+^, creating the necessary concentration gradient between the intra- and extracellular media, leading to the efflux of H^+^ and lactate from muscle during exercise (Robergs et al., 2004). An increase in muscle-released lactate concentrations has been previously observed, suggesting that elevated alkalosis accelerates lactate transport (Maughan et al., 2018). The variations in post-exercise blood pH, lactatemia, and HCO_3_^-^ concentration in STY^ALK^ support the hypothesis of an additive buffering effect of the dietary (ALK) and water (STY) strategies, but not sufficient to cause ergogenic effects during the present exercise modality.

One of our initial hypotheses was that the consumption of bicarbonate-rich water could compensate for the effects of an increased acid load induced by the consumption of an acidizing diet on the acid-base balance. Urinary pH in STY^ACI^ was significantly higher than PLA^ACI^ (STY^ACI^: 6.86 ± 0.60 vs. PLA^ACI^: 6.19 ± 0.45; *p* = 0.01), underlining the significative effect of drinking bicarbonate-rich water on urine alkalinization. Although we did not find significant effects on blood biomarkers, the consumption of mineral water rich in HCO_3_^-^ ions appears to reduce the acid load induced by the acidizing diet by regulating the acid-base balance.

In contrast to previously reported literature, the strategies implemented here did not influence performance with an alkalinizing diet (Limmer et al., 2018), chronic sodium bicarbonate intake (McNaughton and Thompson, 2001) or acute intake of bicarbonate-rich water (Rieux and Duvallet, 2000). In the study by McNaughton and Thompson (2001), which used a similar experimental design to ours, albeit with reduced ecological validity, bicarbonate supplementation in a capsule form resulted in improved anaerobic performance. Most notably, the quantity of bicarbonate administered was 3 to 4 times higher than in our study. Practically, achieving comparable levels of bicarbonate intake via bicarbonate-rich water ingestion would not be possible as participants would be required to drink 6 to 7 L of water daily. Additionally, using the same experimental water as STY in our study, Rieux and Duvallet (2000) reported reduced muscle fatigability at the end of the endurance test. Two major points differentiate this study from ours, the timing of intake and the nature of the test (single vs. repeated). Indeed, in the study by Rieux and Duvallet (2000) water was not ingested chronically but only on the day of testing, starting 2 h before the first exercise and lasting until the last test. The second difference lies in the test, either single exercise as in our study or repeated exercise. Data from the Rieux and Duvallet’s (2000) study suggest that drinking bicarbonate-rich water throughout the duration of repeated exercise might be of interest. Recent findings confirm that repeatedly ingesting a small amount of sodium bicarbonate improves repeated all-out performance when ingestion is spread throughout exercise (Dalle et., 2019).

More recently, Hadzic et al. (2019) reported large inconsistencies in the sodium bicarbonate supplementation literature with artificial dietary supplements with a higher dose of bicarbonate compared to this study, showing that effects on performance could differ even when the same exercise and supplementation protocols were applied (Saunders et al., 2014). Saunders et al. (2014) did not show any relationship between the magnitude of change in circulating biomarkers related to acid-base balance, such as bicarbonate and pH, and subsequent enhancement in performance of HI exercise of similar duration as in the present study. Also, as stated in the study of Thomas et al. (2021), there is considerable inter-individual variability in humans in the assimilation of an exogenous alkalinizing intake and its buffering effect. This might have contributed to the fact that we did not find any effect on performance despite the change in acid-base balance biomarkers.

Water naturally rich in bicarbonate ions can be utilized to supply the necessary bicarbonate ions for optimizing buffering capacity. Our study indicates that the consumption of bicarbonate-rich water over several days positively modifies acid-base balance. In practical terms, a daily intake of 1.5 to 2 l of bicarbonate-rich water as part of a hydration plan could gradually enhance buffering capacity in the days leading up to competition. It is recommended to initiate this strategy at least three days before the competition and distribute water intake throughout the day, such as 0.5 l in the morning, 0.5 l around noon (between meals), in the afternoon, and possibly in the evening. This approach could be particularly beneficial for athletes aiming to avoid excessive supplement consumption or who experience gastrointestinal issues (McNaughton et al., 2016 ). As highlighted in our study, an alkaline diet enhances the beneficial effect of bicarbonate-rich water on acid-base balance. Just like with hydration, athletes can optimize their diet by predominantly including fruits and vegetables in an alkaline-based diet. However, it is strongly recommended to test these nutritional strategies for each athlete during a training period or a non-competitive phase to prevent discomfort associated with dietary modifications. While this mode of consumption did not improve anaerobic performance, future research can explore whether the combination of chronic and acute intake of bicarbonate-rich water could enhance performance. Further studies could investigate the effects of these nutritional strategies, particularly in non-weight-bearing sports, among non-recreational athletes (Froio de Araujo Dias et al., 2015), and in the context of repeated exercise, especially when bicarbonate-rich water is consumed throughout the exercise duration. Future studies should take into account sodium supplementation, which could also have an impact on anaerobic performance (Durkalec-Michalski et al., 2023). Finally, one potential limitation of this study was the use of a basic randomization method, based on random division without taking into account the pre-visit outcomes. Indeed, participants were not matched based upon the primary outcomes of the previsit, for instance, the familiarization performance test, before being randomly assigned to one of the two dietary groups. Finally, considering that participants were recreationally trained men, we may hypothesize that the nutritional strategy implemented in our study could have enhanced effects on athletes with a higher level of performance. Indeed, following a high-intensity exercise, metabolic acidosis is more pronounced in elite athletes compared to athletes at the regional level (Hanon et al., 2011). Consequently, supplementing with bicarbonate-rich water along with an alkalizing diet could optimize the buffering capacity of more trained athletes.

## Conclusions

In conclusion, this study was the first to investigate the influence of chronic consumption of bicarbonate-rich water on the regulation of acid-base balance and anaerobic performance and its combined effects with an alkalizing or an acidizing diet. Despite the implementation of these nutritional strategies, bicarbonate-rich water intake did not affect anaerobic performance, regardless of dietary intake. While we did not measure blood HCO_3_^-^ concentration and pH before the warm-up, we showed that the chronic consumption of bicarbonate-rich water significantly impacted resting urinary pH irrespective of alkalizing or acidizing dietary intake. Furthermore, we observed significantly greater post-exercise peak blood lactate in response to bicarbonate-rich water consumption compared to the placebo condition. In addition, blood pH and bicarbonate concentration were affected by both bicarbonate-rich water and alkalinizing diet during the recovery period compared to the placebo condition. Our results suggest that consuming mineral water rich in HCO_3_^-^ ions potentiates an alkalizing diet's beneficial effects on the biomarkers reflecting acid-base balance during a 1-AO. Finally, consuming bicarbonate-rich water reduces the acid load induced by an acidizing diet.

## References

[ref1] Brancaccio, P., Limongelli, F., Paolillo, I., D'Aponte, A., Donnarumma, V., & Rastrelli, L. (2012). Supplementation of Acqua Lete® (Bicarbonate Calcic Mineral Water) improves hydration status in athletes after short term anaerobic exercise. Journal of the International Society of Sports Nutrition, 9(1), 35. 10.1186/1550-2783-9-35 22835267 PMC3423013

[ref2] Caciano, S., Inman, C., Gockel-Blessing, E., & Weiss, E. (2015). Effects of dietary acid load on exercise metabolism and anaerobic exercise performance. Journal of Sports Science & Medicine, 14(2), 364–371. 25983586 PMC4424466

[ref3] Carr, A., Hopkins, W., & Gore, C. (2011). Effects of acute alkalosis and acidosis on performance: A meta-analysis. Sports Medicine, 41(10), 801–814. 10.2165/11591440-000000000-00000 21923200

[ref4] Chycki, J., Golas, A., Halz, M., Maszczyk, A., Toborek, M., & Zajac, A. (2018). Chronic ingestion of sodium and potassium bicarbonate, with potassium, magnesium and calcium citrate improves anaerobic performance in elite soccer players. Nutrients, 10(11), 1610. 10.3390/nu10111610 30388775 PMC6266022

[ref5] Chycki, J., Kurylas, A., Maszczyk, A., Golas, A., & Zajac, A. (2018). Alkaline water improves exercise-induced metabolic acidosis and enhances anaerobic exercise performance in combat sport athletes. Plos One, 13(11), e0205708. 10.1371/journal.pone.0205708 30452459 PMC6242303

[ref6] Chycki, J., Zając, T., Maszczyk, A., & Kurylas, A. (2017). The effect of mineral-based alkaline water on hydration status and the metabolic response to short-term anaerobic exercise. Biology of Sport, 34(3), 255–261. 10.5114/biolsport.2017.66003 29158619 PMC5676322

[ref7] Deriemaeker, P., Aerenhouts, D., Hebbelinck, M., & Clarys, P. (2010). Nutrient based estimation of acid-base balance in vegetarians and non-vegetarians. Plant Foods for Human Nutrition (Dordrecht, Netherlands), 65(1), 77–82. 10.1007/s11130-009-0149-5 20054653

[ref8] Durkalec-Michalski, K., Kamińska, J., Saunders, B., Pokrywka, A., Łoniewski, I., Steffl, M., & Podgórski, T. (2024). Does sodium bicarbonate based extra-cellular buffering support reduce high intensity exercise-induced fatigue and enhance short-term recovery assessed by selected blood biochemical indices?. Biology of Sport, 41(1), 17–27. 10.5114/biolsport.2024.125591 38188117 PMC10765444

[ref9] Edge, J., Bishop, D., & Goodman, C. (2006). The effects of training intensity on muscle buffer capacity in females. European Journal of Applied Physiology, 96(1), 97–105. 10.1007/s00421-005-0068-6 16283370

[ref10] Froio de Araujo Dias, G., da Eira Silva, V., de Salles Painelli, V., Sale, C., Giannini Artioli, G., Gualano, B., & Saunders, B. (2015). Consistencies in responses to sodium bicarbonate supplementation: A randomised, repeated measures, counterbalanced and double-blind study. Plos One, 10(11), e0143086. 10.1371/journal.pone.0143086 26574755 PMC4648485

[ref11] Grgic, J., Grgic, I., Del Coso, J., Schoenfeld, B., & Pedisic, Z. (2021). Effects of sodium bicarbonate supplementation on exercise performance: an umbrella review. Journal of the International Society of Sports Nutrition, 18(1), 71. 10.1186/s12970-021-00469-7 34794476 PMC8600864

[ref12] Hadzic, M., Eckstein, M., & Schugardt, M. (2019). The Impact of Sodium Bicarbonate on Performance in Response to Exercise Duration in Athletes: A Systematic Review. Journal of Sports Science & Medicine, 18(2), 271–281. 31191097 PMC6544001

[ref13] Halz, M., Kaszuba, M., Helbin, J., Krzysztofik, S., Suchanecka, A., & Zajac, A. (2022). Beta-alanine supplementation and anaerobic performance in highly trained judo athletes. Balt J Health Phys Act, 14(2): Article1. 10.29359/BJHPA.14.2.01.

[ref14] Hanon, C., Rabate, M., & Thomas, C. (2011). Effect of expertise on postmaximal long sprint blood metabolite responses. Journal of Strength & Conditioning Research, 25(9), 2503–2509. 21804425 10.1519/JSC.0b013e3182001807

[ref15] Hanon, C., Thomas-Junius, C., & Giroux, C. (2019). *Sports à haute intensité: mieux comprendre la performance pour mieux l'entraîner*. Institut national du sport, de l'expertise et de la performance.

[ref16] Heil, D. P. (2010). Acid-base balance and hydration status following consumption of mineral-based alkaline bottled water. Journal of the International Society of Sports Nutrition, 7, 29. 10.1186/1550-2783-7-29 20836884 PMC3161391

[ref17] Lancha Junior, A. H., Painelli V. de S., Saunders, B., & Artioli, G. G. (2015). Nutritional strategies to modulate intracellular and extracellular buffering capacity during high-intensity exercise. Sports Medicine, 45 Suppl 1, S71–S81. 10.1007/s40279-015-0397-5 26553493 PMC4672007

[ref18] Lattier, G., Millet, G. Y., Martin, A., & Martin, V. (2004). Fatigue and recovery after high-intensity exercise. Part II: Recovery interventions. International Journal of Sports Medicine, 25(7), 509–515. 10.1055/s-2004-820946 15459831

[ref19] Limmer, M., Eibl, A. D., & Platen, P. (2018). Enhanced 400-m sprint performance in moderately trained participants by a 4-day alkalizing diet: A counterbalanced, randomized controlled trial. Journal of the International Society of Sports Nutrition, 15(1), 25. 10.1186/s12970-018-0231-1 29855319 PMC5984464

[ref20] Maughan, R., Burke, L., Dvorak, J., Larson-Meyer, D., Peeling, P., Phillips, S., Rawson, E., Walsh, N., Garthe, I., Geyer, H., Meeusen, R., van Loon, L., Shirreffs, S., Spriet, L., Stuart, M., Vernec, A., Currell, K., Ali, V., Budgett, R., Ljungqvist, A., & Engebretsen, L. (2018). IOC consensus statement: dietary supplements and the high-performance athlete. International Journal of Sport Nutrition and Exercise Metabolism, 28(2), 104–125. 10.1123/ijsnem.2018-0020 29589768

[ref21] McNaughton, L., Gough, L., Deb, S., Bentley, D., & Sparks, S. (2016). Recent developments in the use of sodium bicarbonate as an ergogenic aid. Current Sports Medicine Reports, 15(4), 233–244. 10.1249/JSR.0000000000000283 27399820

[ref22] Mc Naughton, L., & Thompson, D. (2001). Acute versus chronic sodium bicarbonate ingestion and anaerobic work and power output. Journal of Sports Medicine and Physical Fitness, 41(4), 456–462. 11687764

[ref23] Ragone, L., Guilherme Vieira, J., Camaroti Laterza, M., Leitão, L., da Silva Novaes, J., Macedo Vianna, J., & Ricardo Dias, M. (2020). Acute Effect of Sodium Bicarbonate Supplementation on Symptoms of Gastrointestinal Discomfort, Acid-Base Balance, and Performance of Jiu-Jitsu Athletes. Journal of Human Kinetics, 75, 85–93. 10.2478/hukin-2020-0039 33312297 PMC7706673

[ref24] Remer, T., & Manz, F. (1995). Potential renal acid load of foods and its influence on urine pH. Journal of the American Dietetic Association, 95(7), 791–797. 10.1016/S0002-8223(95)00219-7 7797810

[ref25] Richard, R., Jimenez, L., Duvallet, A., & Rieu, M. (2000). Effect of bicarbonated salt water in physiological exercise adaptations. Science & Sports, 15(1), 18–25.

[ref26] Robergs, R., Ghiasvand, F., & Parker, D. (2004). Biochemistry of exercise-induced metabolic acidosis. American Journal of Physiology. Regulatory, Integrative and Comparative Physiology, 287(3), R502–R516. 10.1152/ajpregu.00114.2004 15308499

[ref27] Rosenthal, R. (1994). Parametric measures of effect size. In H. Cooper & L. V. Hedges (Eds.), *The handbook of research synthesis* (pp. 231–244). Russell Sage Foundation.

[ref28] Saunders, B., Sale, C., Harris, R. C., & Sunderland, C. (2014). Sodium bicarbonate and high-intensity-cycling capacity: variability in responses. International Journal of Sports Physiology and Performance, 9(4), 627–632. 10.1123/ijspp.2013-0295 24155093

[ref29] Steffl, M., Kinkorova, I., Talar, K., Jandova, T., Moulisova, K., Omcirk, D., & Petr, M. (2021). The Effects of High Mineral Alkaline Water Consumed over Three Consecutive Days on Reaction Time Following Anaerobic Exercise–A Randomized Placebo-Controlled Crossover Pilot Study. Journal of Human Kinetics, 78, 111-119. 10.2478/hukin-2021-0046 34025869 PMC8120973

[ref30] Thomas, C., Delfour-Peyrethon, R., Dorel, S., & Hanon, C. (2022). Positive Effects of Pre-exercise Metabolic Alkalosis on Perceived Exertion and Post-exercise Squat Jump Performance in World-Class Cyclists. Journal of Strength and Conditioning research, 36(9), 2602–2609. 10.1519/JSC.0000000000003855 33651728

